# Renal and Lung Cysts in Birt-Hogg-Dubé Syndrome: A Continuum of the Same Disorder

**DOI:** 10.7759/cureus.18878

**Published:** 2021-10-18

**Authors:** Jad A Degheili, Bassem Tanios, Mouhamad Nasser

**Affiliations:** 1 Division of Pediatric Urology, Department of Surgery, Children's Hospital of Eastern Ontario, University of Ottawa, Ottawa, CAN; 2 Division of Urology, Department of Surgery, American University of Beirut Medical Center, Beirut, LBN; 3 Division of Nephrology and Hypertension, Department of Internal Medicine, American University of Beirut Medical Center, Beirut, LBN; 4 Department of Respiratory Medicine, National Coordinating Reference Centre for Rare Pulmonary Diseases, Louis Pradel Hospital, University Hospital of Lyon, Lyon, FRA

**Keywords:** pneumothorax, renal cell carcinoma, syndrome, birt-hogg-dubé, lung cysts, renal cysts

## Abstract

Birt-Hogg-Dubé (BHD) syndrome is a rare autosomal-dominant disorder, affecting multiple organs, mostly the skin, lungs, and kidneys. The prevalence of BHD syndrome is difficult to define given the rarity of the disease. Patients present most often with primary spontaneous pneumothorax. Renal tumors are a characteristic finding in BHD, and are often bilateral and multifocal and of the chromophobe and oncocytoma variant. Very scarce reports have highlighted the presence of simple renal cysts, as the only phenotypical renal manifestation, in BHD patients. Herein, we highlight two novel cases of bilateral multiple renal and pelvic cysts, in two females with genetically proven BHD syndrome, doubting a potential association with BHD syndrome.

## Introduction

Birt-Hogg-Dubé (BHD) syndrome is a rare autosomal-dominant disorder, affecting multiple organs, mostly the skin, lungs, and kidneys [1]. The prevalence of BHD syndrome is difficult to define given the rarity of the disease; but it is estimated that 5-10% of patients presenting with primary spontaneous pneumothorax is due to BHD syndrome [2,3]. Renal tumors are a characteristic finding in BHD, and present in 15-30% of cases with a mean age of onset at fourth to fifth decade of life, younger than in the general population [4]. There is a seven-fold increase in developing renal malignancies. Kidney lesions in BHD are most often bilateral and multifocal [4].

Although the utmost majority of patients with BHD-associated kidney diseases present with kidney tumors, very scarce reports have highlighted the presence of simple renal cysts, as the only phenotypical manifestation for kidneys, in BHD patients [5,6]. Herein, we would like to highlight two new cases of bilateral multiple renal cysts, in two females with genetically proven BHD syndrome, doubting a potential association with BHD syndrome.

## Case presentation

**Figure 1:**
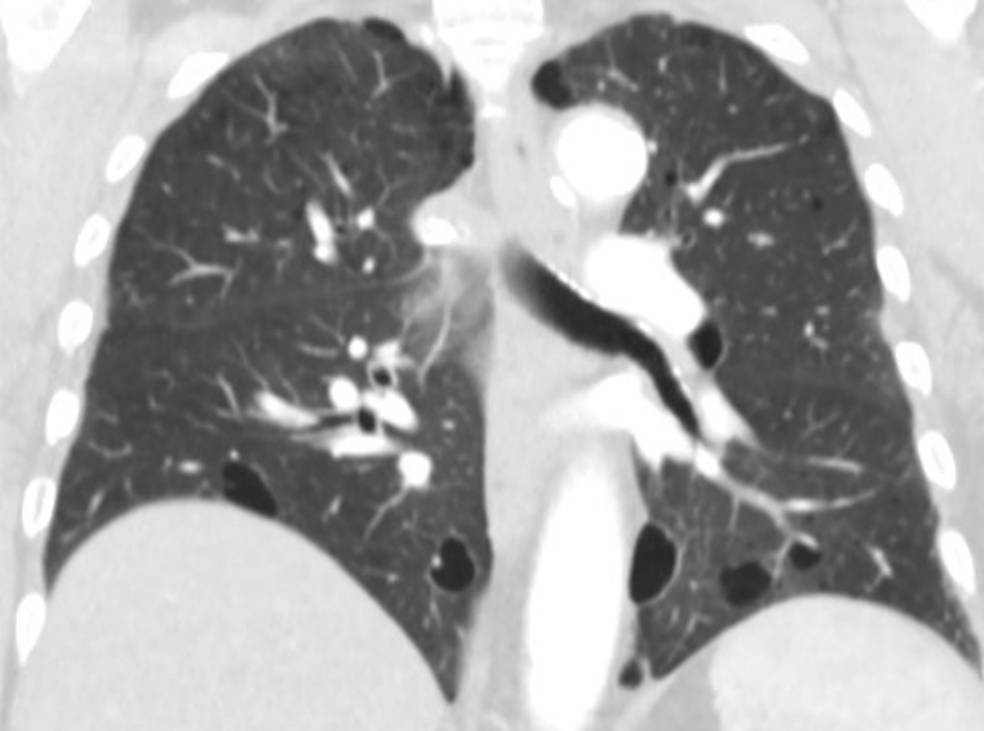
Enhanced computed tomography (CT) scan of the chest (coronal view) demonstrating the presence of bilateral thin wall elliptical-shaped cysts, in the middle and lower lobes of both lungs, with no other ancillary findings.

The first case was a 70-year-old woman, referred for in-depth investigations for bilateral lung cysts. Patient had innumerable small white-to-flesh colored, dome-shaped skin bumps on her face and upper torso. Chest CT demonstrated bilateral thin wall elliptical-shaped cysts with no ancillary findings, in the middle and lower lobes, respectively (Figure 1).

Skin biopsy revealed prominent epidermal strands originating from central hair follicles, suggestive of fibrofolliculomas. FLCN genetic testing was conducted to confirm BHD syndrome. The following mutation, c.1528G>T, p.Glus10-, NM144997.6, within the Folliculin gene was noted. 

**Figure 2:**
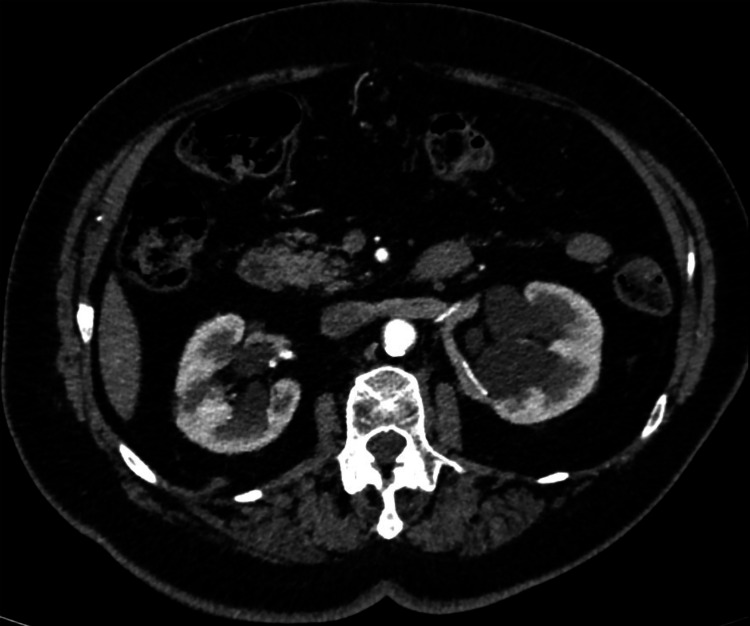
Axial cross section of an abdominal enhanced computed tomography (CT) scan showing bilateral non-enhancing multiple cortical and para-pelvic simple renal cysts. The largest is a para-pelvic left kidney cyst, measuring 5.3 cm in its greatest dimension. No calcifications or septations noted within those cysts.

To rule out renal tumors, an enhanced abdominal computed tomography (CT) showed bilateral non-enhancing rounded cortical and para-pelvic cysts, with imperceptible wall, in both kidneys, without apparent calcifications or septations. The renal cysts were considered simple (Bosniak I classification), and surveillance was not warranted. The largest cyst was para-pelvic, measuring 5.3 cm in greatest dimension (Figure 2).

After four years of follow-up, lung and renal cysts remained stable, with no newly developed cysts. 

**Figure 3:**
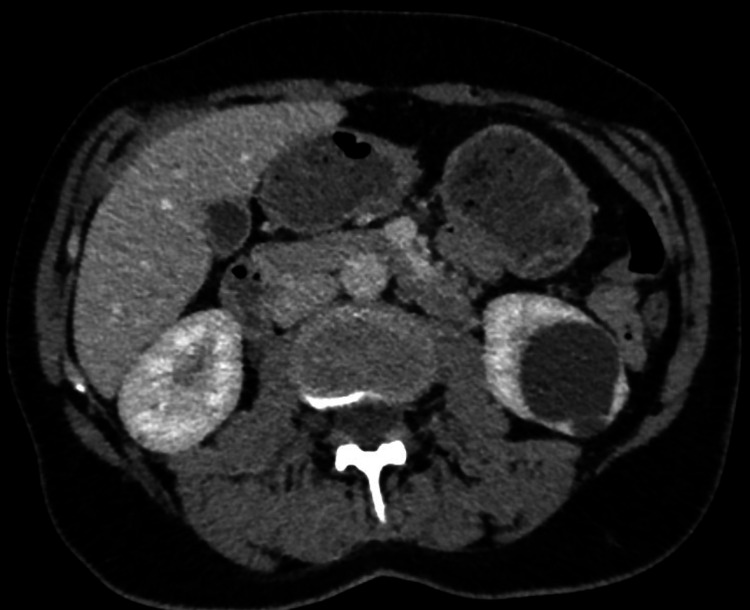
An axial cross-sectional enhanced abdominal computed tomography (CT) scan in a patient genetically proven to have BHD syndrome, showing the presence of a large left non-enhancing simple renal cyst measuring 3.5 cm x 5 cm.

The second case was a 50-year-old woman, never smoker, with a personal and family history of recurrent pneumothoraces. Patient had multiple innumerable characteristic skin lesions on her face and torso. BHD syndrome was suspected in this patient, and confirmed by genetic testing of the Folliculin gene, showing mutation c.1458de1, p.lle486Metfs-5, in exon 13. Chest CT identified bilateral multiple basal elliptical lung cysts. Abdominal CT was also ordered to rule out renal malignancies and instead revealed multiple liver and bilateral renal cysts. Renal cysts were benign-looking, without septations or calcifications. The largest was of 3.5 x 5 cm² (Figure 3).

After five years, the lung, liver, and kidney cysts remained stable in size and number.

## Discussion

Initially reported in 1977, Birt et al. described the BHD syndrome as a familial dermatological disorder with constellation of skin fibrofolliculomas and multiple pulmonary cysts with resultant recurrent pneumothoraces [1]. Nearly 80% of patients with BHD syndrome have multiple lung cysts seen on CT scans of the chest [2].

BHD syndrome is caused by a germline mutation in the FLCN gene located on chromosome 17p11.2, which encodes the tumor suppressor protein, Folliculin [3]. Folliculin is a tumor suppressor protein involved in the regulation of adenosine monophosphate-activated protein kinase and in the mammalian target of rapamycin signaling pathways [7]. The prevalence of BHD syndrome is difficult to estimate given its rarity; but around 5-10% of patients presenting with primary spontaneous pneumothorax are found to be diagnosed with BHD syndrome [3]. 

The risk of developing renal tumors is in fact seven-fold more in BHD patients, compared to the general population. Kidney lesions in BHD are most often bilateral and multifocal, with the oncocytoma and chromophobe variant being the most common malignancy (>50%) [4].

Skolnik et al., in their largest family cohort of patients with BHD syndrome, found renal cysts in eight out of 31 patients (26%). The mean age at diagnosis was 41 years [5]. Similarly, Toro et al. reported bilateral renal cysts in two out of 13 patients (15%), with 71 and 41 years of age [6]. Finally, Liu et al. found bilateral renal cysts in four out of 22 patients with BHD syndrome (18%). The cysts were bilateral in all cases with a mean age at diagnosis of 51 years [8]. In a recent study of 2095 healthy volunteers, simple renal cysts were observed on abdominal ultrasonography in 49 individuals (2.3%). Only four had bilateral renal cysts, and all were male. Multiple renal cysts were found in six participants [9].

Renal cysts are prevalent in general population, with a prevalence between 5.2% and 41%, according to age group and imaging modalities, with CT or magnetic resonance imaging being the most sensitive [10]. Older age, male sex, hypertension, and smoking history, all increase the likelihood for developing renal cysts [11]. Therefore, whether the presence of renal cysts, in particular, is an incidental finding, or indeed a true phenotypic feature of BHD syndrome, in a way similar to renal tumor, is yet to be determined.

## Conclusions

BHD syndrome is a rare disorder, with multiple manifestations involving mostly the skin, lungs, and kidneys. Renal cell carcinomas are well known to be associated with BHD. Yet, few case series have extrapolated the renal manifestations into just the presence of simple renal cysts, of no malignant potentials. In the general population, renal cysts are commonly isolated, unilateral, and simple, whereas multiple and bilateral renal cysts are less frequent. Therefore, in patients with BHD, the presence of bilateral and multiple renal cysts may raise questions about causality. Nevertheless, causality cannot be attributed to BHD syndrome outside large-case control studies. As such, further description and investigation of renal cysts, in BHD syndrome, is eagerly needed and warranted.
